# Protocol for a randomized placebo-controlled clinical trial using pure palmitoleic acid to ameliorate insulin resistance and lipogenesis in overweight and obese subjects with prediabetes

**DOI:** 10.3389/fendo.2023.1306528

**Published:** 2024-01-19

**Authors:** Ecesu Cetin, Brian Pedersen, Lindsey M. Porter, Gail K. Adler, Mehmet Furkan Burak

**Affiliations:** ^1^ Division of Endocrinology, Diabetes and Hypertension, Brigham and Women’s Hospital and Harvard Medical School, Boston, MA, United States; ^2^ Sabri Ulker Center, Department of Molecular Metabolism, Harvard T.H. Chan School of Public Health, Boston, MA, United States

**Keywords:** palmitoleic acid, obesity, prediabetes, overweight, insulin resistance, fatty liver

## Abstract

Palmitoleic acid (POA), a nonessential, monounsaturated omega-7 fatty acid (C16:1n7), is a lipid hormone secreted from adipose tissue and has beneficial effects on distant organs, such as the liver and muscle. Interestingly, POA decreases lipogenesis in toxic storage sites such as the liver and muscle, and paradoxically increases lipogenesis in safe storage sites, such as adipose tissue. Furthermore, higher POA levels in humans are correlated with better insulin sensitivity, an improved lipid profile, and a lower incidence of type-2 diabetes and cardiovascular pathologies, such as myocardial infarction. In preclinical animal models, POA improves glucose intolerance, dyslipidemia, and steatosis of the muscle and liver, while improving insulin sensitivity and secretion. This double-blind placebo-controlled clinical trial tests the hypothesis that POA increases insulin sensitivity and decreases hepatic lipogenesis in overweight and obese adult subjects with pre-diabetes. Important to note, that this is the first study ever to use pure (>90%) POA with < 0.3% palmitic acid (PA), which masks the beneficial effects of POA. The possible positive findings may offer a therapeutic and/or preventative *pathway* against diabetes and related immunometabolic diseases.

## 1 Introduction

Obesity primarily manifests as a package of immunometabolism diseases that includes insulin resistance, diabetes, fatty liver disease, and atherosclerosis. All these diseases share similar adipo-centric lipid derangements and immunometabolic underpinnings. High carbohydrate and high (poor quality) fat diets contribute to the pathogenesis of obesity and its related complications. Adipose tissue is one of the most important endocrine organs in these processes. Adipose tissue is a safe and efficient energy storage site that shows extreme plasticity when handling excessive caloric intake. However, after a certain threshold, it gets inflamed and contributes to obesity-related disease states (1, 2). During the initial stages of energy surplus, it stores extra calories via increasing *de novo* lipogenesis inside the adipose tissue. Lately, our lab discovered that it is simultaneously secreting some lipokines as signaling molecules to crosstalk with distant organs such as the liver and the muscle. Lipokine signaling decreases *de novo* lipogenesis in the liver, improves insulin sensitivity and increases glucose uptake in the muscle (3). Expectedly, a persistent calorie excess with overfeeding during obesity trumps this signaling rescue mechanism and leads to ectopic fat accumulation.

**Figure 1:**
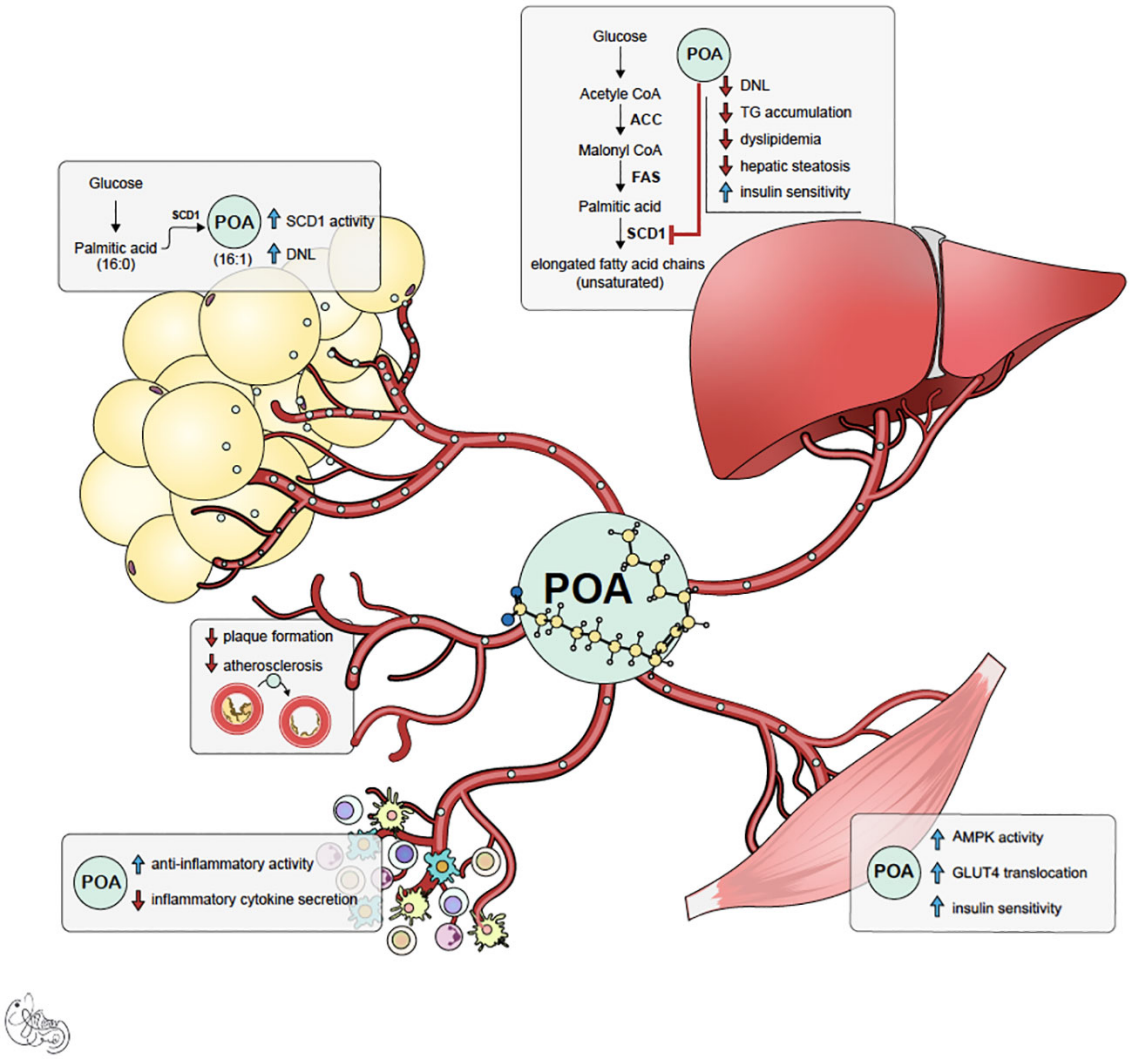
Summary of potential mechanism of actions of palmitoleic acid (POA). POA is synthesized both in adipose tissue and liver by desaturase enzyme SCD-1. However, it is likely that POA synthesized in subcutaneous adipose tissue (lipokine) is healthier signal and has beneficial pleiotropic effects.

Palmitoleic acid (POA) is one of the crucial components of this rescue mechanism. Acting as a lipokine, POA is secreted from adipose tissue and has beneficial pleiotropic effects in distant organs (3, 4) (**Figure 1**). POA is a nonessential, monounsaturated omega-7 fatty acid with 16 carbons (C16:1n-7). POA can be obtained from dietary sources such as macadamia nuts, dairy, sea buckthorn oil, and certain fish. It can also be synthesized via the desaturation of palmitic acid (PA, C16: 0) primarily in adipose tissue and liver by a delta-9 desaturase called stearoyl-CoA desaturase-1 (SCD1). Like other fatty acids, POA contributes to complex lipids, including triglycerides, phospholipids, and cholesterol esters, and circulates as a free (non-esterified) fatty acid. The actions of POA tend to oppose those of palmitic acid (PA), which is known to induce ER stress, inflammation, apoptosis of healthy cells, insulin resistance, glucose intolerance, and steatosis in the liver and muscle (3–20). The beneficial effects of POA have been observed in obesity-related immunometabolic diseases, including insulin resistance, diabetes, fatty liver disease, and atherosclerosis. In cellular and animal studies, POA supplementation has been shown to improve overall glucose metabolism and increase whole-body insulin sensitivity (10, 11, 21–28) via increased muscle and adipose tissue glucose uptake by enhancing GLUT content and AMPK activation (5, 29, 30). This prevented weight gain (11, 22, 31, 32) improved beta-cell function, and prevented palmitic acid-induced beta-cell death (6, 8). Additionally, POA attenuated inflammation (16, 17, 33–40) by decreasing circulating pro-inflammatory cytokines and inflammatory markers in various tissues. Furthermore, it prevented atherosclerosis by decreasing inflammasome activation and organelle stress (9). Consistent with these data, POA improved the circulating lipid profile and ameliorated fatty liver disease by increasing lipogenesis in safe storage sites (adipose tissue) and decreasing lipogenesis in pathological or toxic lipid storage sites (muscle and liver) (3, 41–43).

Fat storage in the liver contributes to insulin resistance, leading to fatty liver disease (NAFLD), and may progress to steatohepatitis (NASH) and finally cirrhosis. Consistent with animal studies, human studies have shown that higher POA is correlated with lower insulin resistance, diabetes incidence, dyslipidemia, hypertension, atherosclerosis, and myocardial infarction (36, 44–60). The positive association studies were followed by an extensive meta-analysis of 16 multicentric prospective cohort studies. The meta-analysis showed that higher levels of POA were associated with a lower risk of developing type 2 diabetes (61). Many interventional human studies are focused on dyslipidemia, and they showed that POA supplementation (mainly through macadamia nut oil) reduced serum LDL cholesterol and triglycerides and increased HDL cholesterol (47–51). However, some of the interventional studies failed to show significant beneficial effects (62, 63). This is most likely because these studies administered POA supplements that contained a significant amount of palmitic acid, which can mask the beneficial effects of POA and has numerous detrimental effects on its own. Consequently, there is a necessity to address this knowledge gap in the literature and test pure exogenous POA administration on insulin sensitivity and serum lipid profiles. This randomized, placebo-controlled study will address this paucity of knowledge regarding pure POA’s effects. The primary aim of this study is to determine the beneficial effect of POA supplementation on insulin sensitivity, measured by the gold standard test: the hyperinsulinemic-euglycemic clamp, in overweight and obese subjects with prediabetes. Secondary aims address the amelioration of hepatosteatosis, whole-body fat mass, serum lipids and inflammatory markers.

## 2 Methods and analysis

### 2.1 Study design and setting

**Figure 2:**
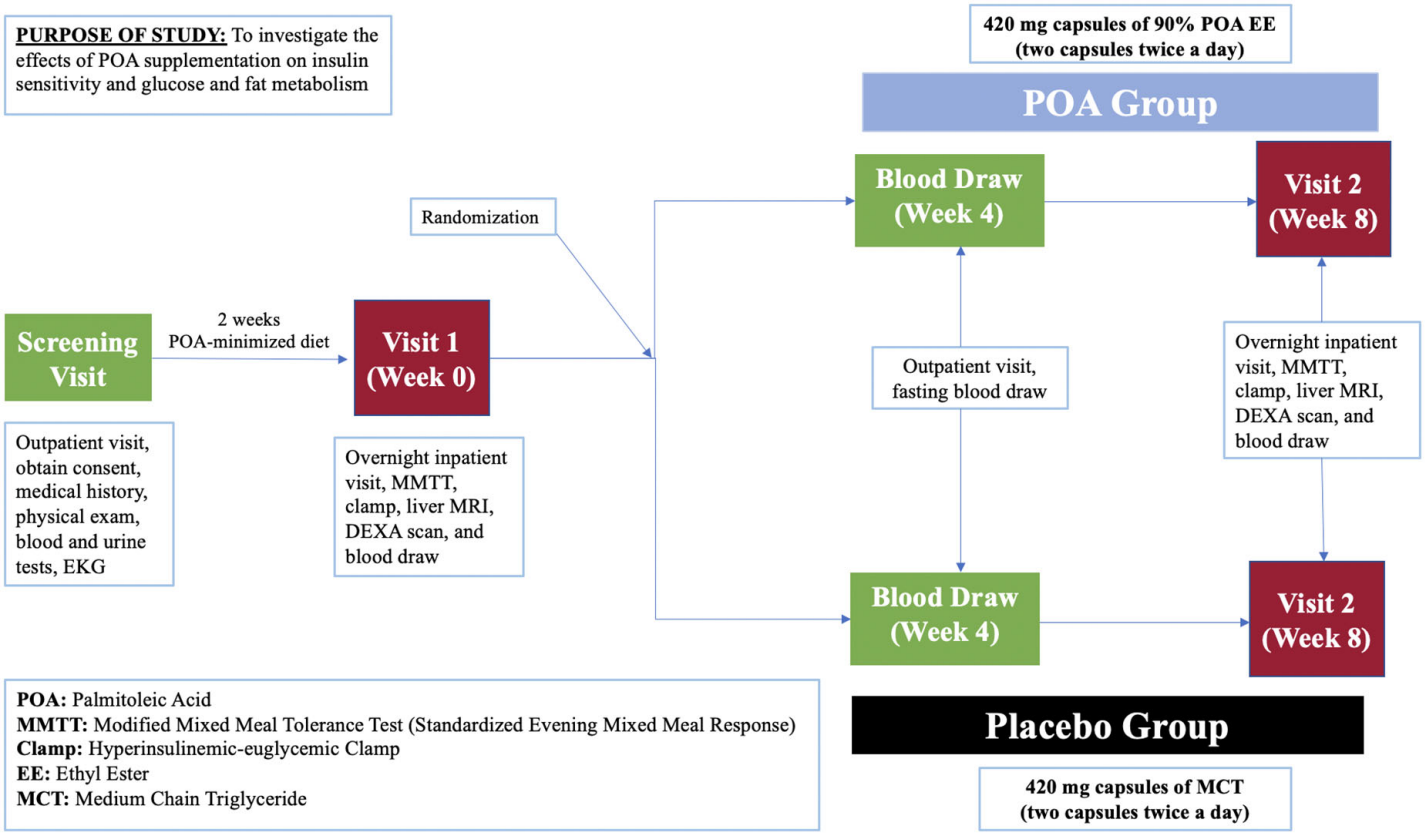
Schedule of the study procedures.

This is a prospective, single-center, 8-week, double-blind, randomized, placebo-controlled clinical trial (NCT05560971) enrolling 40 individuals with a prediabetes and BMI of 25-40 kg/m^2^. Study participants will be recruited at Brigham and Women’s Hospital in Boston, Massachusetts, USA with the inclusion and exclusion criteria provided in **Table 1**. **Figure 2** shows a description of the enrollment and evaluation procedures. The protocol of this study was approved by the Institutional Review Board of Mass General Brigham. This investigator-initiated study is funded by Tersus Life Sciences, LLC, protocol no: 2022P001764.

**Table 1:** Eligibility criteria.

**Inclusion Criteria**	Age 18 to 70 years with BMI 25-40 kg/m^2^
	Overweight and obese individuals with insulin resistance, prediabetes and/or impaired glucose tolerance HbA1c between 5.6-6.5, Impaired fasting plasma glucose (>99, ≤126 mg/dL), or HOMA-IR value above 2.5.
		BP <150/90 with or without medication, GFR>60, ALT, AST <300, TSH within normal ranges with or without medication
	**Exclusion Criteria**		Pregnancy or breastfeeding
			Use of any medications (except thyroid hormone with normal TSH, anti-hypertensives with blood pressure <150/90, and rescue inhalers for asthma)
		Use of OTC supplements (except vitamin D), avoiding supplements containing lipid supplements (e.g., fish oil macadamia oil, cod liver oil, krill oil, flaxseed, sea buckthorn oil) within 3 months of study participation is ensured	
Greater than 3 servings/day combined of cheese, milk, kefir, or yogurt for the last 3 months before the study	
Diagnosed with any type of diabetes mellitus and/or taking glucose-lowering medications	
Recent weight loss (more than 7% of TBW in last 3 months)	
Established major chronic diseases such as major cardiovascular disease (history of myocardial infarction, stroke, heart failure, coronary artery bypass graft, arrhythmia, peripheral arterial disease), bleeding disorder or anticoagulation use, active cancer, end-stage renal disease, proteinuria (>3g/day), dementia, severe chronic obstructive pulmonary disease (needs systemic steroid therapy), significant liver disease (ALT-AST>300)	
History of ongoing smoking cigarettes >1 pack/day, alcohol abuse, or drug abuse	
Treatment with any investigational drug in the one month preceding the study	

### 2.2 Subject enrollment

Participants will be recruited from the greater Boston area (using local news media, MBTA advertising, flyers, poster advertisements, brochure mailings, and online resources), Brigham and Women’s Hospital (using flyers), and the Mass General Brigham Health Care System (using Rally and Patient Gateway programs). Patient Gateway Research Invitations allow for direct communication with any eligible subjects who have not opted out of receiving Research Invitations. Additionally, potentially eligible participants who previously consented to be contacted about future research studies will be contacted (via email or phone).

Potential participants will undergo a 2-hour in-person screening visit to determine if they meet study eligibility requirements. Screening visit laboratory measurements are specified in **Table 2**. Written informed consent will be obtained during the screening visit by a licensed study physician. The screening visit will include a complete medical history and physical examination, laboratory studies (complete blood count, comprehensive metabolic panel, urine analysis, insulin, TSH, and a pregnancy test), and an electrocardiogram (EKG). Randomization to either POA supplement or placebo will be stratified based on BMI and biological sex and it will be done by the BWH Investigational Drug Pharmacy Service. The investigational agent (POA supplement) and placebo will be dispensed in a similar shape, size, color, and odor. For both POA and placebo, participants will be instructed to take 4 capsules daily: 2 capsules in the morning and 2 capsules in the evening before meals at consistent times each day. To facilitate timely medication intake, we send morning and evening reminder text messages to participants every day. Each 420 mg capsule of POA is 90% pure POA. Therefore, each capsule will contain 378 mg of POA, and the total amount of pure POA consumed per day in the treatment arm will be 1512 mg.

**Table 2:** Screening visit tests.

Screening Visit Tests	
Blood Tests	Urine Tests
Complete Blood Count with differentials	Urinalysis, complete
Comprehensive Metabolic Panel	Urine Pregnancy Test
Hemoglobin A1c	EKG
TSH	
DNA Analysis	
Insulin	

### 2.3 Palmitoleic acid-free diet

Two weeks before Inpatient Visit 1 (Week 0), enrolled participants will be asked about their dietary habits and exercise routine. Afterwards, they will be instructed not to change their dietary and exercise habits intentionally except for eliminating foods known to contain large amounts of POA from their diet until after the entire study is complete. These foods are sardines, menhaden, anchovies, pollock, herring, macadamia nuts, sea buckthorn oil, krill oil, cod liver oil, and macadamia nut oil. Also, they will be limiting their dairy intake to no more than 1 total dairy serving a day. Our goal is to eliminate any residual effect of POA present in participants’ regular diets and to improve the detection of the acute effects of POA. With the exception of having participants avoid POA rich foods, we aim to achieve a real-life experience with no dietary or exercise coaching. Additionally, participants will be abstinent from activities that alter insulin sensitivity the day before the inpatient visits such as drinking any alcohol, consuming caffeine, and performing strenuous exercise.

### 2.4 Inpatient visits (Week 0 and Week 8)

Participants who qualify and decide to join the study will undergo two overnight study visits at week 0 and week 8 at Brigham and Women’s Hospital. After completing study procedures at week 0, participants will be given the study medication, either placebo or POA, and will be instructed to take 2 capsules twice a day. Laboratory measurements are shown in **Table 3**.

**Table 3:** Admission tests.

Week 0 and Week 8 Assessments	
Admission Labs Day 0	Overnight Fasting Labs with Clamp Blood Sets
Urine HCG (Women)	Complete Blood Count with differentials
Potassium	Comprehensive Metabolic Panel
Hematocrit	Hemoglobin A1c, Insulin, C-Peptide, Glucagon, Cortisol, Melatonin
MMTT Day 0 (7 Hours Food Withdrawal)	hs-CRP, TNFa, IL-6
MMTT 0 Min-Glucose/C peptide	Total Adiponectin, Leptin
MMTT 30 Min-Glucose/C peptide	FABP4, NEFA, Glycerol
MMTT 60 Min-Glucose/C peptide	EDTA Extra Plasma, Extra Serum
MMTT 90 Min-Glucose/C peptide	RNA from PBMC
MMTT 120 Min-Glucose/C peptide	Lipid Panel, Essential Fatty Acid Panel, Direct LDL

#### 2.4.1 Modified mixed meal tolerance test: standardized evening mixed meal response

A mixed meal tolerance test is commonly used in clinical research for the evaluation of insulin secretion, sensitivity and beta cell function by measurements of fasting and postprandial glucose and C-peptide levels after test meal consumption (64).

In our study, after at least a 6-hour food withdrawal, one intravenous line will be placed in the hand/wrist of the participant and used to facilitate frequent blood drawing throughout the study. Participants will ingest a standardized test meal (360 kcal, ∼44% carbohydrate, 35% fat, and 21% protein) composed of an 8-fluid-ounce nutrition supplement drink (BOOST^®^). Blood samples will be collected before the meal ingestion and at 30-minute intervals after the meal for 2 hours for glucose and C-peptide measurements.

#### 2.4.2 Hyperinsulinemic-euglycemic clamp

The hyperinsulinemic-euglycemic clamp is the gold standard method for the determination of insulin sensitivity (65). The principle is an acute elevation of insulin concentration and maintenance at the basal level which would normally cause hypoglycemia. Blood glucose is kept at basal concentrations with variable glucose infusion through negative feedback. This prevents glucose-insulin feedback loop and hypoglycemic neuroendocrine response which interferes with the determination of whole-body insulin sensitivity. Under these steady-state concentrations, hepatic glucose production is suppressed, and the rate of glucose infusion is equivalent to whole-body glucose uptake. Insulin sensitivity is denoted as M/I where M reflects the rate of glucose disposal measured by glucose infusion and I stands for insulin concentration (66).

In our study, one intravenous line will be placed in an arm and used to facilitate frequent blood drawing throughout the day after an overnight fast. This line will again be placed in a “Hot Box” (a heated box set at 150F). The second intravenous line, which will be inserted into the antecubital vein, will be used for infusions of dextrose and insulin. They will receive a low-dose primed continuous infusion of insulin (20 mIU/m2/min) for two hours from ~ 8:00 AM to ~10:00 AM, followed by a high-dose primed insulin infusion (120 mIU/m2/min) from ~10:00 AM to ~12:00 PM. Dextrose (20%) will be infused to maintain blood sugar at ∼90 mg/dL throughout the infusion of insulin. A priming insulin dose will be given at the time of each insulin infusion change. Insulin infusion rates were chosen to partially suppress hepatic glucose production during the low-dose infusion, while completely suppressing hepatic glucose production and maximally stimulating peripheral glucose utilization during the high-dose infusion in insulin-resistant participants. Plasma glucose levels will be measured every 5 minutes with a bedside glucose analyzer (YSI). Our goal is for each participant to experience four hours of blood glucose levels at 90 mg/dL. In addition, finger-sticks may be performed to assess the participant’s glucose levels using a standard bedside glucometer (only in-case of unexpected YSI or IV line problems). Clamp Blood Sets are displayed in **Table 4**. During this procedure, the participant will be asked how they feel at regular intervals using a standardized questionnaire. We will also measure the participant’s blood pressure and heart rate before beginning the clamp.

**Table 4:** Clamp blood sets.

Clamp Blood Sets	
T=0, Blood Set 1	T=115, Blood Set 2
Cortisol, Comprehensive Metabolic Panel, Insulin, C-Peptide, hsCRP, Lipid Panel, Direct LDL	Cortisol, Insulin
Complete Blood Count with differentials	EDTA Extra Plasma
Hemoglobin A1c	Low Dose Insulin Infusate
Fatty Acid Panel, Essential	T=240, Blood Set 3
TNFa, IL-6, Total Adiponectin, Leptin	Cortisol, Insulin
RNA from PBMC	EDTA Extra Plasma
FABP4, Melatonin, NEFA, Glucagon	High Dose Insulin Infusate
Glycerol	
EDTA Extra Plasma	
Extra Serum	
Q 5 min Glucose monitoring begins	

#### 2.4.3 Potassium supplementation

The day before the hyperinsulinemic clamp, participants will have their plasma potassium level checked upon admission to the Inpatient Research Unit. If potassium levels are less than 4.0 mmol/L, the participant will be given potassium orally. The dose of potassium will vary depending on the amount of potassium needed to increase plasma potassium to ≥ 4.0 mmol/L.

#### 2.4.4 Magnetic resonance imaging

Nonalcoholic fatty liver disease is characterized by the presence of steatosis, which is considered the histopathologic hallmark of this condition. The aberrant accumulation of iron is frequently observed in individuals diagnosed with diffuse hepatopathies and metabolic disorders. Non-invasive imaging methods, not limited by sampling error, observer variability, and decreased patient acceptance, are of increasing importance in the diagnosis and monitoring of liver disease (35). MRI is widely accepted as the best technique for quantification of liver fat. MRI for proton density fat fraction (MRI-PDFF) liver is a commonly used reference standard that only requires one breath hold for fat quantification and whole-organ imaging (36). MRI-PDFF is best suited for when the drug or intervention has a high likelihood of an anti-steatotic effect. Study participants will complete an abdominal MRI scan without contrast. More specifically, the procedure is a liver iron quantification MRI for proton density fat fraction. It will occur at the standard Radiology Facility on the 1.5-Tesla Siemens Aera MRI machine. The participant will lie flat in the scanner for approximately 40 minutes to complete the MRI.

#### 2.4.5 Dual energy X-Ray absorptiometry

DEXA, the gold standard method for body composition on a molecular level, provides an evaluation of fat mass, non-bone lean mass, and bone mineral content. Body composition will be measured by DEXA at week 0 (baseline) and week 8. This is an accurate, validated method with minimal radiation exposure. A Discovery-W DEXA (Apex software version 4.0, Hologic, Bedford, MA), a clinical and research scanner located in the BWH outpatient facility, will be used to measure total body fat percentage and detailed analysis of fat mass locations and volumes. Despite a low dose radiation exposure, pregnant participants will not be allowed to participate in the study (67–70).

#### 2.4.6 Sleep monitoring

Since sleeping time and the sleep-wake cycle effects insulin sensitivity measures, subjects will wear an Actiwatch Respironics^®^ wristwatch, which monitors their sleeping time and quality for their overnight visit (71). We will examine their sleep quality and schedule. If we find atypical sleep patterns, we will also use sleeping quality/time as a co-variate for the final analysis.

### 2.5 Week 4 visit

After four weeks on the study medication, participants will come to the Center for Clinical Investigation (CCI) for a blood draw. Participants will be asked to fast after midnight before the morning blood draw. Laboratory measurements are specified in **Table 5**.

**Table 5:** Week 4 tests.

Blood Draw (Week 4) Study Tests	
Complete Blood Count with differentials	FABP4, NEFA, Glycerol
Comprehensive Metabolic Panel	EDTA Plasma
Hemoglobin A1c, Insulin, C-Peptide, Glucagon	RNA from PBMC
hs-CRP, TNFa, IL-6	Lipid Panel, Standard Triglycerides Total Cholesterol HDL Cholesterol LDL Cholesterol (Direct) Cholesterol/HDL Ratio (Calculated), Non-HDL Cholesterol (Calculated)
Total Adiponectin, Leptin	

### 2.6 Statistical analysis

#### 2.6.1 Statistical methods

We will use a paired t-test for the primary endpoint as we measure insulin sensitivity M and M/I values from the same individuals before and after taking POA vs placebo. However, given the relatively small sample size of the study, which raises the possibility of results not being normally distributed, we will also use Wilcoxon non-parametric test to compare two paired groups (before and after, POA vs placebo). The focus of these analysis will be insulin sensitivity; however, we will use multivariate regression analysis, which also provides the flexibility of controlling for and evaluating covariates such as age, sex, sleep, and BMI. We will apply the same testing to all endpoints. According to some association studies, we expect to see a higher magnitude of the POA effect in women. We will aim for study enrollment to be at least 50% women to be able to detect sex differences in the POA effect.

#### 2.6.2 Sample size and power

The study is powered only for the primary endpoint, insulin sensitivity. We expect a mean insulin sensitivity-M value (measured as steady state clamp glucose infusion rate) of approximately 7.5 ± 1.7 mg/kg/min with placebo, based on previous clamp studies^30^. Based on previous human studies, we expect to see at least a 20% difference between placebo vs. POA treatments. A sample size of 40 subjects with complete data will provide greater than 80% power for these assumptions. We will perform stratified randomization based on subjects’ BMI (<30>), and gender for homogenous treatment groups. 3:2 POA vs placebo assignment will allow us to achieve this randomization goal. We were also implementing oral glucose tolerance test results for the additional stratification of the randomization, however additional invasive testing before starting the trial period caused scheduling conflicts and compliance problems. Hence, we have discontinued OGTT for the stratification criteria.

#### 2.6.3 Data management plan

The study will utilize REDCap (Research Electronic Data Capture), a free, web-based application designed to support data capture for research studies. The database will be hosted on secure, password-protected servers. The database is fully HIPAA compliant and provides audit capabilities. All study data will be inputted into REDCap by study staff and the platform provides automated export procedures for seamless data downloads to Excel and common statistical packages.

## 3 Anticipated results

### 3.1 Expected outcome

The primary endpoint of this study is insulin sensitivity which will be evaluated by the hyperinsulinemic euglycemic clamp and estimated by the HOMA-IR. The secondary endpoints demonstrated in **Table 6** are glucose and c-peptide measurements performed after the modified mixed meal tolerance test, liver fat quantification, total body fat mass and body composition evaluated by DEXA scan, serum levels of fasting glucose, insulin, c-peptide, liver function tests, fasting lipid panel, hsCRP, circulating inflammatory cytokines (TNF-a, IL1-B, IL-6), FABP4, glucagon, POA, free fatty acids, glycerol, adiponectin, and leptin. We expect POA compared to placebo to improve insulin sensitivity, glucose tolerance, hepatosteatosis, whole-body fat mass, serum profile of lipids, and inflammatory markers. Additionally, we anticipate a higher therapeutic impact of POA in women compared to men.

**Table 6:** Outcomes.

**Primary Endpoint**	Insulin sensitivity, evaluated by hyperinsulinemic euglycemic clamp (gold standard test), and estimated by the HOMA-IR
**Secondary Endpoints**	Liver fat quantification (evaluated by liver MRI-PDFF)
	Modified mixed meal tolerance test (standardized evening mixed meal response)
	Total body fat mass and body composition (will be evaluated by DEXA scan)
	Serum; fasting glucose, insulin, C-peptide, LFTs, fasting lipid panel, hsCRP, circulating inflammatory cytokines (TNFa, IL1-B, IL6), FABP4, glucagon, POA, free fatty acids, glycerol, adiponectin, leptin, and cortisol.

### 3.2 Benefits

Successful completion of the study will lead to a better understanding of the effects of POA on glucose and lipid metabolism. Understanding the effects of POA on insulin sensitivity and lipogenesis has the potential for a profound public health impact in the fight against obesity and type 2 diabetes. Possible positive results of this study might create a new dietary approach to prevent and/or treat diabetes and related complications such as fatty liver disease. High-quality unsaturated fatty acids (such as omega-3) have been shown to improve dyslipidemia and improved the lives of people with cardiovascular disease (72). Testing the effect of omega-7 fatty acids like POA will increase the understanding of how high-quality fatty acids could be beneficial for insulin resistance and prediabetes in addition to dyslipidemia. There will be no direct benefit to the subject for taking part in this study.

### 3.3 Pitfalls and alternative approaches

This is an 8-week clinical trial, and the duration might not be sufficient to observe the beneficial effects of POA on insulin sensitivity. Because other studies demonstrate the effects of POA on metabolism after 1 month, we decided to limit the study timeframe to 8 weeks to make sure patient compliance is established (27, 47). The study is powered for the insulin sensitivity measured with the gold standard method of the hyperinsulinemic-euglycemic clamp. Therefore, 40 participants may not be adequate to exhibit statistically significant differences in secondary endpoints being liver fat mass, total body fat mass, body fat composition, glucose and c-peptide levels post-mixed meal, serum glucose, insulin, c-peptide, liver function tests, fasting lipid panel, hs-CRP, circulating inflammatory cytokines (TNF-a, IL1-B, IL-6), FABP4, glucagon, POA, free fatty acids, glycerol, adiponectin, leptin measurements. Additional studies designed to focus on secondary endpoints might be necessary to show significant effects. We aim to select participants who has insulin resistance via using criteria identifies individuals with overweight-obesity and prediabetes. However, we might still get participants with high insulin sensitivity, where there might not be enough therapeutic window to improve. In that case, we might need to add more strict inclusion and/or exclusion criteria or randomize potential insulin sensitive participants equally to treatment arms.

## 4 Discussion

Following the accumulation of evidence from animal studies, several positive association studies have demonstrated the correlation of POA with decreased insulin resistance and diabetes incidence. A study with 100 Caucasian non-diabetic, overweight volunteers with first-degree type 2 diabetic relatives undergoing 9 months of lifestyle intervention demonstrates that circulating POA is a strong and independent determinant of insulin sensitivity measured by hyperinsulinemic-euglycemic clamp tests, implying its critical role in the pathophysiology of insulin resistance in humans (46). Another study performed with non-diabetic participants has shown that plasma POA is an independent determinant of glucose tolerance, insulin sensitivity, and beta cell function in non-diabetics. This underscores POA’s key role in protecting systemic glucose metabolism from the detrimental effects of excess NEFA and adiposity (44). After the improvements in glucose metabolism in preclinical models and association studies, an extensive longitudinal study was performed. Data from 16 prospective studies with 63,682 participants without known diabetes at baseline and 15,180 participants who developed cases of type 2 diabetes over the average of 9 years and over up to 20 years of follow-up were analyzed. POA was measured as a biomarker of dairy fat consumption. This large meta-analysis shows that higher POA levels are associated with a lower risk of developing Type 2 diabetes mellitus (61). Yet, some association studies claimed a positive correlation between obesity and POA and interpreted it as a negative impact (73–85). Despite the claims of others, we think that the increased levels of POA are due to its role as a rescue mechanism of the body against gluco-lipotoxicity. *In vitro* experiments done in human islet cells supports this idea by demonstrating that POA ameliorates the detrimental effects of palmitic acid by lowering high glucose-induced apoptosis of beta cells (7). Several human interventional studies focusing on the effects of a POA-enriched diet on dyslipidemia have been carried out. For instance, 0.75 ml/day sea buckthorn seed oil (4.89% POA) supplementation for 30 days prompted a 27% decrease in LDL cholesterol in hypertensive individuals (86). Furthermore, the administration of 15% of daily caloric intake with macadamia nuts (high in POA) for 4 weeks resulted in a 5.3% decrease in LDL cholesterol, a 3% reduction of total cholesterol, and a 7.9% increase in HDL cholesterol levels in hypercholesterolemic men (48). Additionally, a study comparing a Macadamia nut rich diet (42.5-gram POA/day) with an Average American Diet showed an 8.9% decrease in LDL cholesterol and a 9.4% decrease in total cholesterol (50). However, some of the studies failed to show beneficial effects. Perhaps this is because these studies administered POA supplements that contained a significant amount of palmitic acid which can mask the beneficial effects of POA. In summary, association studies could be misleading both positive and negative ways according to many confounders, which is hard to control. Thus, there is a critical unmet need to assess the causal effects of POA directly and blindly (un-biased) on human glucose and lipid metabolism.

While designing translational studies, one of the most important aspects is safety. Unsaturated fat, namely POA, has beneficial effects on glucose metabolism, whereas the same amount of saturated fat, namely palmitic acid, is detrimental (22, 44). This concept has been proven by investigating the dietary habits of Greenland Eskimos in the 1970s. Their diet consisted of 55% fat enriched with POA (3 times higher than a Western diet, an average of 6.54 grams/day), which surprisingly resulted in significant protection from diabetes, dyslipidemia, and coronary heart disease as compared to Eskimos living in Denmark, who were consuming a Western diet (87, 88). POA is the second most abundant monounsaturated fatty acid within the standard American diet (89). The average Western diet consists of approximately 2 grams of POA (90). Human intervention studies have documented the consumption of up to 15.3 g of POA/day for the duration of up to four weeks, with no serious adverse effects reported (48). According to an expert panel, 5 g POA/d (as 10 g of either Provinal® EE or TG) for the average user and 10 g POA/d (as 20 g of either Provinal® EE or TG) for a 90th percentile user is Generally Recognized as Safe (GRAS) (91). 1512 mg of pure palmitoleic acid will be given to the treatment arm in our study. The available evidence suggests that consumption of POA within the specified intake levels does not result in any significant adverse effects.

To date, there are no clinical trials that have assessed the impact of supplementation with pure POA versus placebo on glucose tolerance and insulin resistance in humans. The proposed randomized, placebo-controlled study will address this gap in knowledge, and give us an opportunity to possibly translate our basic science discovery to humans. Here we hypothesize that we can mimic the rescue mechanism of adipose tissue against obesity by supplementing high dose POA exogenously, which will improve whole-body insulin sensitivity and ameliorate hepatosteatosis.

## Ethics statement

The studies involving humans were approved by Mass General Brigham institutional review board. The studies were conducted in accordance with the local legislation and institutional requirements. The participants provided their written informed consent to participate in this study.

## Author contributions

EC: Investigation, Writing – original draft, Writing – review & editing. BP: Investigation, Writing – review & editing, Project administration, Data curation, Formal analysis. LP: Investigation, Project administration, Writing – review & editing. GA: Investigation, Project administration, Writing – review & editing, Conceptualization, Methodology, Supervision, Formal analysis, Resources, Data curation. MB: Conceptualization, Investigation, Methodology, Project administration, Supervision, Writing – review & editing, Data curation, Formal analysis, Funding acquisition, Resources, Validation, Visualization, Writing – original draft.
